# Finding confidence and inner trust as a parent: experiences of group-based compassion-focused therapy for the parents of adolescents with mental health problems

**DOI:** 10.1080/17482631.2019.1684166

**Published:** 2019-10-29

**Authors:** Anna Sofia Bratt, Idor Svensson, Marie Rusner

**Affiliations:** a Faculty of Health and Life Sciences, Department of Psychology, Linnaeus University, Växjö, Sweden; b Child & Adolescent Psychiatric Clinic, Södra Älvsborg Hospital, Region Västra Götaland, Borås, Sweden; c Institute of Health and Care Sciences, Sahlgrenska Academy, University of Gothenburg, Gothenburg, Sweden; d Department of Research, Region Västra Götaland, Södra Älvsborg Hospital, Borås, Sweden

**Keywords:** Compassion-focused therapy (CFT), parenting, adolescent mental health, qualitative research, lifeworld research, group therapy

## Abstract

**Purpose**: Compassion-focused therapy (CFT) can alleviate the stress and challenges faced by the parents of adolescents with mental health (MH) problems. Although CFT interventions have shown promising results, few studies have examined its effectiveness in adolescent psychiatric settings. Therefore, this study examined the participant experiences of group-based CFT for the parents of adolescents with MH problems.

**Methods**: The reflective lifeworld research (RLR) approach was used to conduct in-depth interviews with eleven parents, focusing on participant experiences of group-based CFT. Meaning-oriented data analysis was undertaken.

**Results**: The essential meaning of the phenomenon of participating in group-based CFT was understood as *finding confidence and inner trust as a parent*, characterized by an understanding of one’s own needs, which provided parents with the confidence to support their children. The phenomenon is further explicated with its three constituents: (a) taking care of oneself and one’s child; (b) being open and sharing experiences; and (c) acceptance and hope for the future.

**Conclusions**: The CFT intervention enabled parents to find their agency and strengthened their relationships with their children. The findings underscore the need to acknowledge the supportive role parents play in the recovery of children who receive psychiatric care.

Caring for a child who needs additional support because of mental health (MH) problems is a challenging experience for parents. Emotional issues are a normal part of the parenting process; however, the additional burden that is associated with MH problems of a child can be emotionally distressing and taxing to parents. Parents of adolescents with MH problems face higher levels of frustration, uncertainty, and interpersonal conflict, and they may feel that they are criticized, blamed, and treated differently by family and friends (Moses, 2010). There is a lack of studies that have investigated the experiences of parenting an adolescent with MH problems (Brown, 2018; Jivanjee, Kruzich, & Gordon, 2009). This is important because a young person’s MH outcomes may depend on his or her parent’s response (Jorm, Wright, & Morgan, 2007). Parental strain refers to the negative consequences of caregiving responsibilities (e.g., financial strain, fatigue, sadness, guilt, and parental stress; Brannan & Heflinger, 2006). Research on the caregiver strain of parents whose children have mental illnesses is scarce, especially when compared to studies that have focused on caring for children with chronic illnesses, adult children with MH problems, and older adults (Mendenhall & Mount, 2011). Self-stigma is defined as the internalization of stigmatizing views that are held by other people (e.g., negative views about mental illness or the tendency for parents to blame themselves for their child’s problems) and it exacerbates parental strain (Hasson-Ohayon et al., 2014). The severity of children’s MH problems is the factor that most strongly influences parental strain (Angold et al., 1998; Vaughan, Feinn, Bernard, Brereton, & Kaufman, 2013; Wang & Anderson, 2018). Furthermore, parents with pre-existing MH issues experience greater levels of parental burden than their mentally healthy counterparts (Angold et al., 1998). In addition, parents of adolescents with MH issues are often excluded from their child’s treatment planning process (Jivanjee et al., 2009); this may hinder the support that parents can provide to their child. Traditional approaches to the treatment of child and adolescent MH problems focus on treatment of the presenting symptoms (Brown, 2018; Mendenhall & Mount, 2011) rather than on the role of the parents as a resource that can positively influence the child. Further, most studies that have included parents in their research design have focused primarily on parental satisfaction with the treatment that is provided to the child (Brown, 2018). One exception to this trend is Brown’s (2018) study, whereby she examined parental perceptions of a half-year intense treatment that adolescents had received in an adolescent MH service in Sydney, Australia. Specifically, she examined whether the parents believed that the treatment was instrumental in their child’s recovery, and found a relationship between hope and parents’ sense of agency. Further, hopeful parents also felt important and believed that they had the ability to support their children. Parents also appreciated the opportunity to participate in the treatment, and they were able to independently think, reflect, and make decisions without relying on external help. In contrast, parents who reported low levels of hope after the conclusion of the treatment were less confident of their ability to support their children, and they were disappointed that the experts were unable to “fix” their child. When the therapists tried to help such parents reflect on their own roles, the parents found it difficult to understand the expectations of the professional. According to Brown (2018), treatments that help parents discover their own sense of agency allow them to trust their own wisdom rather than that of the experts. Therefore, interventions that help parents manage their strain and find their own sense of agency benefit not only caregivers but also the children with MH problems.

Compassion-focused therapy (CFT) was developed to help people who experience high levels of shame and self-criticism cultivate a compassionate care orientation towards themselves and others (Gilbert, 2017; Gilbert & Choden, 2013). CFT provides psycho-education on the nature of the evolved human mind, and it integrates interventions that focus on self-compassion. In addition, CFT utilizes a range of specific mind training techniques that are collectively referred to as compassionate mind training (CMT). Exercises include breathing exercises, visualization, and behavioural rehearsals (Gilbert, 2010, 2019). By promoting mindfulness and compassionate ways of attending, thinking, and behaving, the individual increases his or her ability to tolerate, engage with, regulate, validate, and soothe difficult emotions such as anxiety, anger, and shame (Gilbert, 2012). Developing a compassionate inner voice and tone for one’s thoughts and motives can help individuals reduce hostile self-criticism and focus on compassionate correction, growth, and improvement (Gilbert, 2010). Hence, CFT and CMT can benefit parents who struggle with the complex situational demands of caring for a child who needs additional support.

The results of non-randomized clinical trials have found that group-based CFT can benefit individuals who struggle with personality disorders (Lucre & Corten, 2013), dementia (Collins, Gilligan, & Poz, 2018; Craig, Hiskey, Royan, Poz, & Spector, 2018), eating disorders (Gale, Gilbert, Read, & Goss, 2014), and complex MH problems (Gilbert & Procter, 2006; Judge, Cleghorn, McEwan, & Gilbert, 2012). However, qualitative research studies of participants’ perspectives and experiences of group-based CFT interventions are scarce. One exception is Lawrence and Lee (2014), who investigated the experiences of five women and two men who had participated in CFT to address the adverse effects of trauma. Four participants experienced group-based CFT, whereas three received individual CFT. The researchers identified five themes, such as the difficulties involved in silencing the inner critic, the emotional aspects of therapy (e.g., the importance of the therapeutic relationship), and realizing that you are not alone in your struggles. Although participants initially experienced difficulties in developing a kinder attitude towards themselves, they eventually reported a positive emotional experience of self-compassion and a more positive outlook towards the present and future.

In conclusion, parenting a child with MH problems is challenging. One can experience self- and child-focused anxieties (e.g., fear of the future), unwarranted anger, frustration, and exhaustion, as well as a profound sense of sadness about the MH status of one’s child and the inability to “take the pain away”. Shame and self-stigmatization may hinder parents’ caregiving abilities. CFT seeks to help people face their challenging feelings with courage, wisdom, warmth, and care. This can help parents be present in the moment and be attuned to the needs of their children. However, past research studies have not examined whether CFT is helpful to parents in general or specifically to the parents of children with MH problems (Carona, Rijo, Salvador, Castilho, & Gilbert, 2017). Therefore, the aim of the present study was to describe the lived experience of group-based CFT for the parents of adolescents who had been treated in a child and adolescent psychiatric outpatient speciality clinic. The present study is part of a larger research study that examined the perspectives of children and utilized quantitative analysis.

## Method

### The RLR approach

The purpose of reflective lifeworld research (RLR) is to describe a phenomenon in depth, which in this case is participant experiences of group-based CFT as reported by the parents of adolescents who have received treatment for MH problems in a child and adolescent psychiatric outpatient speciality clinic. The RLR approach, founded on phenomenology, continental philosophy and a hermeneutic lifeworld approach, strives to describe rather than explain or interpret people’s lifeworlds and everyday experiences (Dahlberg, Dahlberg, & Nyström, 2008). To capture all nuances of the phenomenon of interest, the researcher must “bridle” the natural tendency “to know how things are” (Dahlberg & Dahlberg, 2019). Instead, the researcher needs to be present, and pay attention with an open mind; reflecting and restraining from letting theoretical or personal assumptions influence the process of understanding. In the slow bridled process of finding the structure of meanings in the lived variations of the phenomenon the common essential characteristics are revealed (Dahlberg, 2006). The essential characteristics of the phenomenon constitute the essence, which capture the common thread of the participants´ experiences. The different aspects of the phenomenon are referred to as the constituents, and they are illustrated with quotations. The essence and the constituents together describe what it means to participate in group-based CFT for the parents of children with MH problems (Dahlberg et al., 2008).

### Context

The participants of the present study were sampled from a child and adolescent psychiatric outpatient clinic in Region Västra Götaland, Sweden. The clinic provides specialized MH services that address complex MH problems, most of which entail comorbidities. The present study is a part of a larger research study that was conducted among adolescents aged 14–17 years and their parents who had participated in a group-based CFT intervention. Adolescents who had received treatment at the clinic between January 2017 and the autumn of 2018 were requested to participate in a group-based CFT intervention. The parents of consenting adolescents were requested to join the parental group. The CFT intervention was adapted and developed by the first author for use with adolescents with mental illness and their parents, based on the transdiagnostic CFT approach (Gilbert, 2009, 2010). Parallel group sessions were simultaneously conducted for adolescents and parents. The present study focused on parental experiences; adolescents’ experiences will be presented in another article.

### Intervention

The intervention consisted of eight 2-hour group sessions in accordance with the principles of CFT and the needs of the participants. The first phase involved psycho-education about our “tricky brain” and how threatening negative emotions can be understood from an evolutionary perspective (Gilbert, 2010, 2014). The group members were encouraged to reflect on the balance between their three types of affect regulation: threat, drive, and safety. The next phase focused on the need to understand the motivational basis of emotions and how this affect our thoughts and behaviours. The participants engaged in mindfulness- and compassion-based exercises (e.g., soothing breathing, creating a safe place). Additionally, strategies to develop a compassionate self-image and understand the role of self-critique were introduced. The structure and contents of the sessions were the same for parents and adolescents, and they were encouraged to cooperatively complete their homework assignments. A consistent theme that was emphasized during the intervention pertained to the means by which parents must support themselves in order to care for their children.

### Participants and procedure

The parents received oral and written information about the study before they participated in the CFT intervention. In total, five CFT groups were conducted from January 2017 until the autumn of 2018. All parents (n = 28, 17 mothers and 11 fathers) who had completed the CFT treatment were invited to participate in the study, and eleven parents (six women, and five men; age range: 35–57) agreed to join. The reasons for declining participation was not collected. In total, nine interviews were conducted since there where two married couples (i.e., a mother and a father of the same child where interviewed together). All eleven participating parents had a child who was receiving psychiatric care at the clinic. One divorced couple of a 12 year old son participated in the parental group, while the son did not participate in the adolescent group, due too low age. Another divorced father participated in the parental group, without the daughter participating in the adolescent group. Descriptions of each interview and interviewees is presented in Table I.

**Table I.** Description of the participants in each interview.

Where the interviews are coloured, the participants are parents of the same child.

The CFT intervention was delivered by a group leader, a clinical psychotherapist (not one of the authors), who had completed CFT training with the Compassionate Mind Foundation. An assistant who had extensive experience in psychiatry and other related fields (but no specific training in CFT) provided assistance during the group sessions.

### Ethical considerations

The Regional Ethical Review Board in Gothenburg, Sweden granted ethical approval for the present study (Number: 330–16). Confidentiality and protection of the participants’ identities were ensured. Further, all participants provided written consent regarding their participation and use of their data.

### Data collection

The phenomenon “parents´ experiences of group-based CFT” was the focus of the open in-depth interviews, which were organized by the first author. The interviews were conducted at the child and adolescent psychiatric clinic (this setting was chosen by the participants), and each interview lasted for 28–97 minutes. At the beginning of the interview, the participants were informed that the aim of the study was to capture the meanings that they attach to their participation in the CFT intervention rather than their experiences of the contents of the intervention. According to the RLR approach, the interview is an open dialogue that occurs between the interviewer and interviewee without a formal set of questions. The interview began with the following open-ended question: “I am interested in your experience of participating in the CFT intervention. Can you tell me about your experience”? The interviewer encouraged the participants to deeply reflect on their experiences by posing questions such as, “Can you tell me more about it?” and “Can you give an example of your experience?” The interviews were recorded digitally and transcribed verbatim. Parents’ responses were translated from Swedish to English by a professional bilingual translator.

### Data analysis

The first author analysed the transcribed interviews in accordance with the RLR described by Dahlberg et al. (2008). First, the researcher read the interview transcripts several times to familiarize herself with their content. Subsequently, the researcher focused on the parts of the text that were embedded with the units of meaning. Each unit of meaning (i.e., sentences or a longer descriptions of the experience of the phenomenon) was identified in each separate interview transcript by way of marks in the margin. Similar units of meaning were grouped into clusters to develop a structure. This preliminary analysis of the clusters yielded a temporary pattern of meanings and their interrelationships. The focus of the analytic process shifted between the entire transcript and its constituent parts until the explicit units of meanings yielded a more abstract structure of meanings. Finally, the constituents that represented the variations of the essence were described, and relevant quotations from the verbatim transcripts were chosen to illustrate the constituents. The last step of the analytic procedure entailed articulation of the essential meaning structure of the phenomenon, and the unity of the essence and its constituents, which together was referred to as “a new whole”.

## Results

In the following section, the essence is presented first. It demonstrates how participation in a group-based CFT intervention for parents is characterized at a general level. Thereafter, the different aspects of the phenomenon, namely, the constituents, are described and illustrated using quotations (i.e., excerpts of the participants’ responses).

The essential meaning of participation in the group-based CFT intervention for parents is *finding confidence and inner trust as a parent*. An understanding of one’s own needs provides parents with confidence and strength to support the child. Learning to be present in the moment and validating the child’s feelings and experiences, without attempting to fix or control, allow the parents to connect to and communicate with the child. Sometimes, the phenomenon means reflecting upon and thinking about hardships without attempting to avoid pain, and at other times, rejoicing in the child’s recovery. Self-understanding, acceptance, and mutual respect is experienced by interacting with the other group members and by being open and willing to talk about difficulties. Confidence and inner trust as a parent entails a knowing that it is not always possible to cope with the challenges of everyday life; at times, they become overwhelmed and forget to take care of themselves. Indeed, no one is perfect. However, at other times, confidence and inner trust as a parent entail an ability to stay calm, and let things go. The essence of the phenomenon illuminate trust in the ability to care for the child, to set boundaries, and feel proud rather than ashamed of having a child with MH problems.

The following three constituents, represent nuances and unique variations, which further explicate the meaning of the phenomenon: (a) taking care of oneself and one’s child; (b) being open and sharing experiences; and (c) acceptance and hope for the future.

### Taking care of oneself and one’s child

The lived experience of participating in a group-based CFT intervention means acquiring knowledge about body, emotions, and brain. Being aware of the feelings and stop reacting automatically helped parents reflect upon whether their reactions were appropriate or not. *“There’s a reason for it; there’s nothing strange about it. You have to have it. … It’s quite nice, and you can calm yourself down on your own if you know that you’re not going overboard or, like, you just breathe and calm yourself down”.*

When understanding the function of different emotions it was easier to communicate with the child, to validate rather than engage in problem solving. *“The child wants us to listen and not tell her what she should do. We’ve gotten better at that”.*

To participate in group-CFT for parents means learning to take care of oneself. The necessity to care of own needs entailed everyday activities such go for a walk, go to bed on time and eat healthy food. The parents recognized self-care as caring for the child’s well-being too, and refrain from criticizing him/herself as a parent. When the parents were less self-critical, they tended to be less critical towards the child. *“The same goes for not criticizing the kid. Maybe you did it without thinking first; didn’t mean to be critical, but sometimes that’s still how it goes”.*

Participating in a group-based CFT intervention entail a gradual process of self-understanding and a desire to grow as a person. *“Learning to take a step back, to look into this, take care of myself and not only my professional or my parental role. I also have to be myself. Otherwise, I won’t be of much help … So, I’m working on that too … finding new patterns. It’s going to take a while. I end up in the ditch every day, but if I realize, ‘this is what I feel’, then I’ve succeeded. Then, take care of the green circle again, balance this effort. The last years have certainly taken a lot of effort”.*

Managing everyday life means a constant balancing between a focus on the child’s needs and the demands from school and others, between supporting and pushing. In this turmoil of feelings and challenges, the parents tried to take one-step at the time, moment by moment and dividing the problems into smaller parts. *“I didn’t go all the way down that road as you do when you think that your whole life has gone off the rails. Instead, I stopped and thought about it and became a bit calmer. Then, maybe I thought, ‘This is today. If it doesn’t work, we’ll try again tomorrow’”.*

### Being open and sharing experiences

The lived experience of participating in a group CFT intervention means sharing experiences with other parents. When they opened up and talked about difficulties, they realized others had a hard time too. *“It’s also super useful to get to listen, too. Some people’s experiences were identical with my own. So it’s really, really nice to hear other parents who have problems, that you’re not alone, not different in any way. It’s made me open up to others, too. And it’s so helpful, instead of being ashamed that we’re doing the way we are, to talk about it and then you see that loads of people have these problems—but maybe they just haven’t sought help with them.”*

Although some parents had difficulties to show emotions, the group enabled the parents to feel safe and share both laughter and tears. The group leader helped to create an open climate. Some of the parents described feeling safe at once, while others needed time. However, conflicts arose at times when some participants did not get along. On the other hand, there was an effort accepting differences in the group, instead of criticizing. Sometimes it was difficult to balance between the predetermined structure of the session (i.e., the structure that the leader and material brought to the session) and the free dialogue that the group members were encouraged to engage in. When group members choose not to continue in the group or if they missed a session, the other participants wondered why. Disruption of the group process could be frustrating for some participants who expressed it took time to get back on track. After the end of the course, some of the parents kept thinking of the other group members.

Sharing allowed parents to receive support from others; in turn, the parents realized that they could also help others. Self-disclosure allowed parents to both give and receive. Sharing their feelings and talking about previously undisclosed difficult experiences made it easier for parents to communicate with their children. The parents realized that they could be role models for their children, and this possibility made them feel stronger. Further, parents were able to better understand the perspectives of not only other group and family members but also their friends and colleagues. Sharing their coping strategies made the parents feel closer to others. In particular, divorced parents appreciated the joint perspectives that they otherwise did not experience in their daily lives. *“It’s also super useful to get to listen too. Some people’s experiences were identical to my own. So it’s really, really nice to hear other parents who have problems, that you’re not alone, not different in any way. It’s made me open up to others too. And it’s so helpful. Instead of being ashamed that we’re the way we are, talk about it and then you see that loads of people have these problems, but maybe they just haven’t sought help for themselves”.*

Participation in the parental group was intertwined with the child’s participation in his/her group, and thoughts tended to drift towards their children (e.g., “is she okay?”). Some parents said that they shared the content of the intervention with their child and appreciated having received the same tools as the child. *“It’s great and important that we parents get help too. If our daughter had just gotten help, if she’d come with loads of stuff, and then we continue running our race the way we think we’re supposed to do things, it wouldn’t have had the same effect at all. It wouldn’t. That both kids and parents get help, that’s the most crucial thing, I think”.*

Parents mentioned feelings of helplessness when the hardship of their child could not be alleviated, as well as happiness when the child made progress. *“She’s become much more self-confident and accepted that she is who she is. I know that before, she could get up at five o’clock in the morning to straighten her hair and spend an hour doing her makeup. Now she gets up half an hour before school and puts on a hat and goes to school. She doesn’t wear makeup at all, she doesn’t straighten her hair—she accepts that she is who she is. I feel like it’s a huge sign that she’s self-confident”.*

### Acceptance and hope for the future

When feeling safer, calmer and connected, the family climate changed to a more positive atmosphere, even though difficulties still occurred and the child suffered at times. *“We also talked about stopping—how stopping makes you really see things as they are. Like, maybe you should do more of that. And then, maybe, everything won’t look like everything else; maybe you’ll feel that you can really prioritize what’s important. Because when you have this kind of a family situation and a child doesn’t feel good about things, you can’t act like everything is normal. Or what is ‘normal’?”*

Trusting oneself as a parent means to respect own needs, wishes, and decisions without worrying about the opinions of others. *“Before, we often thought about everyone around us. What will they say if our daughter doesn’t go to school and has to stay at home? And we heard, ‘You just have to drive them to school, I would never have done that with my children’ or … What am I doing wrong? Trying to shut it out: ‘I’m not going to listen to this because I know what’s best for us, and that’s what’s most important’. Trying to shut out everyone else’s opinions”.*

To participate in a group-based CFT intervention means seeing things in new perspectives (e.g., “life is not so bad after all”), and not take things as seriously as they previously did. *“It does that; you know somewhere inside yourself that things are going to be okay, but that this is a part of life. Things can’t always be easy either. I guess you have to go with the flow”.*

## Discussion

The aim of the present study was to deepen our understanding of how participation in a group-based CFT intervention is experienced by the parents of adolescents who have been receiving treatment for MH problems. This phenomenon can be understood as *finding confidence and inner trust as a parent*, characterized by self-understanding, acceptance, and mutual respect. Together with the three constituents *taking care of oneself and one´s child, being open and sharing experiences, and acceptance and hope for the future*, the meaning structure of the phenomenon as a whole was described. The CFT intervention enabled parents to discover their sense of agency (i.e., the belief that they can help themselves and their children). Despite their best efforts, MH professionals find it difficult to help their patients and caregivers discover their sense of agency. As Brown (2018) described, reliance on experts rather than on one’s own agency hinders parents’ ability to care for their children. An essential part of CFT (i.e., the therapy as well as the specific intervention that was used in the present study) entails the recognition that the patient is the expert on matters that pertain to their lives (Kolts, 2016). Since parents possess a great deal of knowledge about their children, they are encouraged to trust their ability to care for their children.

The primary focus of the present study was the lifeworld of the parents and the variety of their lived experiences. The intertwining of self-care and caring for one’s child was essential to the analysis of the phenomenon. CFT aims to cultivate a compassionate self that helps the individual to inhibit unhelpful impulsiveness and nurture empathic, wise, and caring behaviours (Gilbert, 2017). In the present study, the parents reported that enhancing self-care helped them be better caregivers to their children. However, contrary to expectations, parents did not mention the term, “the compassionate self”, in their descriptions. Instead, they seemed to have internalized a kind and caring way of relating to themselves as a way of being.

The CFT intervention enhanced parents’ ability to share their feelings with the children; it also helped them communicate better and be more expressive about their emotions. When parents are able to communicate effectively and be present in the moment, they can serve as a secure base to their children, even in difficult situations; according to Siegel (2015), this is a hallmark of secure attachment. Close relationships and social support have been found to be associated with improved psychological and physical well-being (see Bourassa, Ruiz, & Sbarra, 2019, for an overview). Therefore, it is essential to involve parents (e.g., as providers of support) in the treatment that is provided to adolescents with MH problems.

The group format of the CFT intervention enabled participants to reciprocate compassion with the other group members. Gilbert (2017) contended that there are three types of compassion: the compassion that we feel towards others, the compassion that we receive from others, and the compassion that we demonstrate towards ourselves (self-compassion). According to Gilbert (2017), being open to the compassion of others helps us feel safe and perceive others as a resource rather than a threat; therefore, the cultivation of compassion may buffer against the negative effects of self-stigmatization. The feeling of being valued and understood by the other group members and the sense that one is not alone in one’s struggles was one of the positive outcomes of the CFT intervention; this finding is similar to that reported by Lawrence and Lee (2014). Accordingly, Dahlberg (2007) observed, “You understand yourself and who you are in the world in relation to others”.

The confidence and inner trust that parents have in themselves entail a wish to develop as a person and as a parent. New perspectives instil a sense of hope for the future. Parents rely on their own abilities rather than on the opinions of others by paying attention to their own needs, wishes, and decisions. The CFT approach is in accordance with the “lifeworld-led care” that has been described by Dahlberg, Todres, & Galvin (2009), whereby healthcare professionals help people discover their own agency and develop their own strategies to improve their health and well-being. Dahlberg et al. (2009) articulated an existential perspective of well-being, described as a process that entails flow and vitality, as well as a sense of peace with the present and an openness towards the future. Gilbert & Choden (2013) compared development of a compassionate mind to the task of being one’s own doctor; accordingly, one must identify the cause of the pain, and subsequently use wisdom and care to alleviate the suffering. This process of self-compassion involves understanding, acceptance, and action-orientation; that is, “acceptance is an act of courage that calls for wise action” (Gilbert & Choden, 2013).

### Limitations of the present study

The present qualitative study is one of the first to describe parents’ experiences of participation in a group-based CFT intervention in a child and adolescent psychiatric clinic; consequently, it contributes new knowledge to an unexplored field of inquiry. Although different strategies were used to ensure objectivity in the data analysis and the validity of the findings, there is always a risk that the researchers’ biases may have influenced the findings. According to Dahlberg et al. (2008) objectivity in research should be distinguished from objectivism. A researcher should be as open as possible, and “bridle” his/her tendency to understand too fast. However, no researcher can be free from pre-understanding, and by “bridling openness”, the researcher can move between objectivity and subjectivity (Dahlberg & Dahlberg, 2019). In the present study, the first author, who has extensive experience in the field of CFT, conducted the analysis. To ensure that her past experiences in this field did not result in erroneous conclusions, her work was continuously reviewed by the other two authors who are relatively inexperienced in this field. The first author used different strategies to slow down the process such as using a reflective research journal, and an ongoing dialogue with the co-authors.

Transferability of results is essential to research; for this to be possible, the collected data must be rich in content and include significant variation. For this reason, our findings may not be transferable to other contexts or cultures. Furthermore, the participants’ responses appeared to represent the experiences of only those who found the intervention to be enriching. Indeed, participants who encountered difficulties with the CFT intervention did not participate in the interviews. Another related limitation of the present study is that parents who had aversive or threatening encounters with CFT were not included in the sample. Indeed, other studies have reported that participants may experience a fear of compassion (Lawrence & Lee, 2014); however, this phenomenon was not observed in the present study.

## Conclusions

The findings of the present study provide in-depth knowledge about the experience of parents who participated in a group-based CFT intervention. Healthcare professionals find it difficult to help patients and their caregivers accept their challenging situations and be hopeful about the future. In this regard, the results of the present study demonstrate that group-based CFT can help parents discover their sense of agency and gain the confidence to care for their children.

## Clinical implications

- The treatment of MH problems in children and adolescents typically focuses on the presenting symptoms. Parenting a child with MH problems can be challenging; therefore, there is a need for interventions that can enhance parents’ sense of agency and help them feel hopeful about the future.
- Group interventions for parents provide peer support and alleviate their feelings of shame and self-stigmatisation.
